# Vitamin D3 alleviates nonalcoholic fatty liver disease in rats by inhibiting hepatic oxidative stress and inflammation via the SREBP-1-c/ PPARα-NF-κB/IR-S2 signaling pathway

**DOI:** 10.3389/fphar.2023.1164512

**Published:** 2023-05-16

**Authors:** Doha Reda, Gehad E. Elshopakey, Talat A. Albukhari, Samah J. Almehmadi, Bassem Refaat, Engy F. Risha, Hebatallah A. Mahgoub, Mohamed E. El-Boshy, Fatma M. Abdelhamid

**Affiliations:** ^1^ Clinical Pathology Department, Faculty of Veterinary Medicine, Mansoura University, Mansoura, Egypt; ^2^ Department of Haematology and Immunology, Faculty of Medicine, Umm Alqura University, Makkah, Saudi Arabia; ^3^ Laboratory Medicine Department, Faculty of Applied Medical Sciences, Umm Al-Qura University, Makkah, Saudi Arabia; ^4^ Pathology Department, Faculty of Veterinary Medicine, Mansoura University, Mansoura, Egypt

**Keywords:** hepatic steatosis, vitamin D, high-fat diet, insulin resistance, oxidative stress

## Abstract

**Introduction:** Nonalcoholic fatty liver disease (NAFLD) is a chronic disease characterized by fat deposits in liver cells, which can lead to hepatitis and fibrosis. This study attempted to explore the protective effect of vitamin D3 (VitD) against NAFLD.

**Methods:** Adult male albino rats were randomized into four separate groups: the negative control group was fed a standard rat chow; the positive group received a high-fat diet (20%) and 25% fructose water (NAFLD); the VitD control group was intramuscularly treated with VitD (1,000 IU/kg BW) 3 days per week for 10 weeks; and the NAFLD group was treated with VitD therapy. Biochemical and hepatic histological analyses were performed. Hepatic oxidative stress and inflammatory conditions were also studied. Hepatic expression of sterol regulatory element-binding protein 1-c (SREBP-1-c), peroxisome proliferator-activated receptor alpha (PPAR-α), and insulin receptor substrate-2 was analyzed by quantitative real-time polymerase chain reaction.

**Results and discussion:** The NAFLD rats exhibited elevated terminal body weight, hepatic injury markers, dyslipidemia, glucose intolerance, and insulin resistance. Moreover, the NAFLD rats had increased SREBP-1-c expression and reduced PPAR-α and IRS-2 expressions. Histological analysis showed hepatic steatosis and inflammation in the NAFLD group. In contrast, VitD administration improved the serum biochemical parameters and hepatic redox status in NAFLD rats. Also, VitD treatment ameliorated hepatic inflammation and steatosis in the NAFLD group by decreasing the expression of SREBP-1-c and increasing the expression of PPAR-α. Overall, these results suggest that VitD could have a protective effect against NAFLD and its associated complication.

## 1 Introduction

Nonalcoholic fatty liver disease (NAFLD) is a common disease characterized by the presence of triglyceride deposits in more than 5% of hepatocytes, in the absence of excessive alcohol consumption or other documented causes of liver damage (Rahimi and Landaverde, 2013; Shojaei Zarghani et al., 2018). It is divided into two main forms: the non-progressive form, which rarely develops into cirrhosis and is known as nonalcoholic fatty liver disease, and the progressive form, which leads to cirrhosis and hepatocellular carcinoma and is known as nonalcoholic steatohepatitis (NASH) (Loomba and Sanyal, 2013). Since NAFLD is related to obesity and insulin resistance, it is the best-known liver disease that chronically affects people of different ages (Kaufmann et al., 2021). Significant evidence suggests that excessive consumption of high-fat diets and sugar-sweetened beverages is related to the development and progression of NAFLD (Rahimi and Landaverde, 2013; Softic et al., 2016). Additionally, excessive carbohydrate and fat consumption increases the levels of blood sugar and free fatty acids, resulting in excessive neutral lipid deposition in the liver (Zivkovic et al., 2007). The severity of NAFLD can be increased by excessive fructose consumption as it promotes insulin resistance, *de novo* lipogenesis, and the development of an inflammatory, oxidative stress state (Jarukamjorn et al., 2016). Since NAFLD is a critical cause of abnormal liver enzymes, cryptogenic cirrhosis, and liver transplantation, it is important to manage and control NAFLD (Souza et al., 2012; Softic et al., 2016).

Vitamin D is a fat-soluble prohormone that can be formed in the skin after direct exposure to ultraviolet rays or taken with food (Sharifi and Amani, 2019). To achieve its biological activity, a series of sequential biochemical reactions must take place, including 25-hydroxylation in the liver and then 1-hydroxylation in the kidney (Yin et al., 2012). Classically, it has been implicated in calcium and phosphorus hemostasis, but recent studies indicate its role in managing diseases associated with inflammation and oxidative stress in both human and animal models (Sharifi and Amani, 2019). It also plays a role in improving the lipid profile (Faraji and Alizadeh, 2020). Several lines of evidence suggest that VitD can modulate liver inflammation and improve hepatic responsiveness to insulin by binding to its specific receptor in the liver (Barchetta et al., 2017). It has been demonstrated that an active form of VitD can reduce oxidative stress, generation of inflammatory factors, and hepatic fibrosis in NAFLD resulting from a high-fat diet (Liu et al., 2020). It can also alleviate fatty liver disease in an NAFLD rat model by modulating lipid metabolism and/or impeding cell senescence (Yin et al., 2012; Liu et al., 2020). Moreover, VitD intake could reduce NAFLD severity and its risk factors including dyslipidemia and obesity (Sangouni et al., 2019). Therefore, VitD supplementation may be helpful in the prevention of NAFLD by modulating some serum liver function markers, lipid profile, hepatic redox status, selective molecules involved in the inflammatory and antiinflammatory processes, and mRNA expression levels of regulatory molecules, SREBP-1-c, PPAR-α, and IRS-2 involved in lipogenesis, lipolysis, and insulin signaling, respectively.

## 2 Materials and methods

### 2.1 Materials

#### 2.1.1 Chemicals

Cholecalciferol (vitamin D_3_) was brought from Memphis Company for Pharmaceutical and Chemical Industries (Cairo, Egypt) in the form of ampoules (100,000 IU/mL).

#### 2.1.2 Experimental animals

A total of 32 healthy male albino rats (230–250 g) of about 9 weeks of age were procured from the Animal House of Zagazig University. The rats were housed in standard polypropylene cages (four rats per cage) under appropriate conditions of temperature (25°C ± 2°C), humidity (60%–70%), and light (12-h dark/light cycles). They were provided *ad libitum* with a commercial rodent diet and plain water. The experimental protocol was approved by the Ethical Animal Research Committee of Faculty of Veterinary Medicine, Mansoura University, Egypt (Approval No. 2021; M/35).

### 2.2 Methods

#### 2.2.1 Induction of NAFLD

To establish NAFLD, the rats received a high-fat diet (HFD) with 20% fat. The HFD was prepared according to the work of Shin et al. (2016) with some modifications (we used 19 g of butter oil and 1 g of soybean oil as a source of fat instead of corn oil and lard). In addition, 25% fructose solution was added to the drinking water for 10 weeks (about 2.5 months) (high-fat, high-fructose diet).

#### 2.2.2 Animal treatment

After 2 weeks of acclimatization, the experimental animals were assigned to four groups (eight each), detailed as follows:

• Control group: Rats were maintained on standard chow and fructose-free water
• NAFLD group: Rats were fed an HFD and 25% fructose water
• VitD group: Rats received standard chow, fructose-free water, and VitD treatment
• NAFLD + VitD group: Rats received an HFD, 25% fructose water, and VitD treatment

Vitamin D_3_ was diluted in sterile saline and then injected intramuscularly (1,000 IU/kg BW) 3 days per week throughout the experimental period as mentioned by BaSalamah et al. (2018). The body weight of each rat was taken on day 0 and then every week.

#### 2.2.3 Collection and preparation of samples

After the 10th week, experimental rats were injected intraperitoneally with xylazine and ketamine mixture at doses of 10 mg/kg and 50 mg/kg, respectively, and retro-orbital puncture was performed to collect peripheral blood samples. The blood samples were carefully centrifuged at 1,198 × *g* for 10 min to separate the sera. The obtained sera were then used to study some biochemical parameters. After that, hepatic tissue specimens were collected from the decapitated rats and cut into several parts. One part was used to prepare liver homogenates to assess the hepatic redox status and hepatic pro/antiinflammatory status. The protein content in the homogenates was determined, as reported by Bradford (1976). A hepatic specimen of 1 gm was maintained in RNAlater (Quigen, Germany) for gene expression studies. For histological investigation, hepatic tissue sections were formalin-fixed and then stained with hematoxylin and eosin (H&E). In addition, other sections were frozen, fixed, and stained with Oil Red O.

#### 2.2.4 Serum biochemical analysis

Available commercial kits were utilized to evaluate the biomarkers of liver function: alanine and aspartate aminotransferases (ALT and AST, respectively) (Human Diagnostics Worldwide, Wiesbaden, Germany); alkaline phosphatase (ALP) (ELITech, Paris, France); bilirubin (total and direct) (Diamond, Cairo, Egypt); and total protein and albumin (Stanbio Laboratory, TX, United States). A Spinreact kit was used to analyze the serum glucose level, while serum insulin and 25(OH) VitD levels were quantified by enzyme-linked immunosorbent assay using the appropriate kits (Biospes, Chongqing, China, and DiaMetra SRL, Perugia, Italy, respectively). Homeostasis model assessment of insulin resistance (HOMA-IR) was detected according to the work of Matthews et al. (1985).

Different lipid parameters [triglyceride (TG), total cholesterol (TC), and high-density lipoprotein cholesterol (HDL-C)] were estimated using ready-made assay kits (Spinreact, Sant Esteve de Bas, Spain), and exceptionally very-low- and low-density lipoprotein cholesterol (VLDL-C and LDL-C) values were calculated as recorded by Friedewald et al. (1972). The biochemical serum analyses were performed following the manufacturer’s guidelines and measured using a spectrophotometer (BM, Germany, 5010).

#### 2.2.5 Assessment of hepatic redox status and serum total antioxidant capacity

Malondialdehyde (MDA) concentration and the enzymatic activities of both superoxide dismutase (SOD) and catalase (CAT), together with the reduced glutathione (GSH) level, were determined in the hepatic tissue using standard assay kits from Bio-Diagnostic Co. (Cairo, Egypt), according to the instructions in each corresponding pamphlet. Serum total antioxidant capacity (sTAC) was also estimated by using Bio-Diagnostic kits.

#### 2.2.6 Assessment of hepatic pro- and antiinflammatory status

Specific rat ELISA kits purchased from Cusabio (Wuhan, China) were utilized for the determination of the hepatic concentration of nuclear factor kappa β (NF-κβ), whereas R&D Systems ELISA Kits (Minneapolis, MN, United States) were used to measure hepatic interleukin-10 (IL-10) levels, following the protocol in the assay kits.

#### 2.2.7 Assessment of hepatic gene expression

RNA was first extracted from the hepatic tissue following the RNeasy Mini Kit instructions (Qiagen, Hilden, Germany). Then, cDNA was synthesized using RevertAid Reverse Transcriptase Kits from Thermo Fisher Scientific (Massachusetts, United States), following the manufacturer’s instructions. Quantitative RT-PCR was performed to evaluate the relative expression levels of the following genes: sterol regulatory element-binding protein 1-c (SREBP-1-c), peroxisome proliferator-activated receptor alpha (PPAR-α), and insulin receptor substrate-2 (IRS-2). Primer sequences of SREBP-1-c (Ren et al., 2019), PPAR-α (Ding et al., 2014), and IRS-2 (Kanuri et al., 2016) are shown in Table 1. RT-PCR assay was performed using the MX3005P QPCR system. The preparation of the PCR master mix and cycling conditions was performed using the QuantiTect SYBR Green PCR Kit (Qiagen, Germany), following the manufacturer’s instructions. Rat β-actin (Banni et al., 2010) was used as a housekeeping gene, and a comparative Ct method (2^−ΔΔCT^) was used to detect mRNA expression levels, according to the work of Livak and Schmittgen (2001).

**TABLE 1:** Forward and reverse primers for SREBP-1-c, PPAR-α, and IRS-2.

Rat β-actin	F: 5′ -TCC​TCC​TGA​GCG​CAA​GTA​CTC​T-3′
	R: 5′-GCT​CAG​TAA​CAG​TCC​GCC​TAG​AA- 3′
SREBP-1-c	F: 5′-AGG​AGG​CCA​TCT​TGT​TGC​TT- 3′
	R: 5′-GTT​TTG​ACC​CTT​AGG​GCA​GC- 3′
PPAR-α	F: 5′-TCT​GTG​GGC​TCA​CTG​TTC- 3′
	R: 5′-AGG​GCT​CAT​CCT​GTC​TTT​G- 3′
IRS-2	F: 5′-GAA​GCG​GCT​AAG​TCT​CAT​GG- 3′
	R: 5′-GAC​GGT​GGT​GGT​AGA​GGA​AA- 3′

***SREBP-1-c*,** sterol regulatory element-binding protein 1-c; ***PPAR-α*,** peroxisome proliferator-activated receptor alpha; ***IRS-2*,** insulin receptor substrate.

#### 2.2.8 Histopathological assessment of the liver

Liver sections were preserved in 10% formaldehyde solution and then immersed in paraffin. The obtained tissue was sliced into 5 μm-thick tissue blocks that were stained with hematoxylin and eosin (H&E). The obtained blocks were inspected under a light microscope, as mentioned by Kleiner et al. (2005). To assess hepatic lipid deposition, 10-μm frozen liver sections were prepared using a cryostat (LEICA CM 1800), fixed, and then, stained with ORO (Green and Kehinde, 1974). Using the XSZ-107BN microscope (China) and the Apex Minigrab (UK), the obtained sections were randomly photographed and then automatically analyzed using ImageJ (https://imagej.nih.gov/ij).

#### 2.2.9 Data analysis

The outputs were given as means ± SEM. The Statistical Package of Social Services, version 22 (SPSS 22), was used to perform all statistics using ANOVA, followed by Duncan’s multiple-range test to perform the comparisons between the tested groups. Statistical significance was displayed at *p* < 0.05. Positive ORO-stained areas were evaluated by ANOVA, followed by Tukey’s test for group comparison (*p*-value ≤ 0.05), using GraphPad Prism for macOS, version 9.2.0 (283).

## 3 Results

### 3.1 Effect of vitamin D treatment on body weight

**FIGURE 1:**
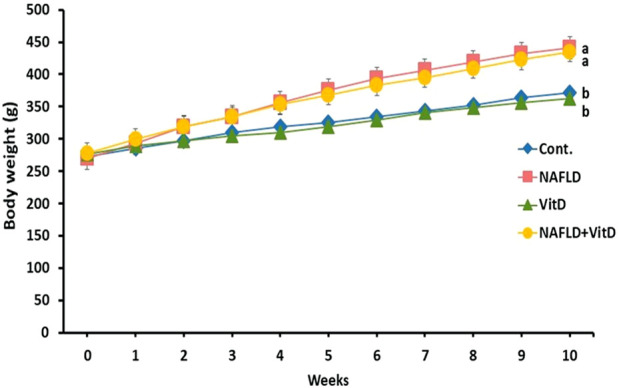
Changes in the body weight (g) of experimental groups during the study period. Data are expressed as means ± SEM, in which those with different superscripts differed significantly (*p* < 0.05). a is used for the high value, while b is used for the lower one. *Cont*., control; *NAFLD,* nonalcoholic fatty liver disease; and *VitD*, vitamin D.

Figure 1 shows that the initial body weights were similar in all the experimental groups. However, a remarkable increase was noted in the terminal body weights of the NAFLD rats compared to the control rats (*p* < 0.05). No substantial variation was observed in the body weight of the NAFLD + VitD compared with the NAFLD group.

### 3.2 Vitamin D treatment improved serum biochemical parameters in NAFLD rats

As shown in Table 2, NAFLD rats showed marked (*p* < 0.05) elevation in the serum ALT, AST, and ALP activities compared to the control rats. Conversely, VitD administration in rats with NAFLD markedly (*p* < 0.05) normalized ALT and ALP serum activities and caused a substantial (*p* < 0.05) decrease in serum AST activity but did not return it to its normal value, suggesting that VitD has hepatoprotective properties. Additionally, the total and direct bilirubin concentrations were pronouncedly (*p* < 0.05) increased in the NAFLD group (Table 2), whereas the total protein and globulin levels were pronouncedly (*p* < 0.05) decreased as opposed to the control rats (Table 2). VitD had no significant (*p* > 0.05) effect on either bilirubin or proteinogram serum results in the NAFLD + VitD group versus NAFLD rats.

**TABLE 2:** Impact of VitD on alterations in the serum liver injury indices, proteinogram, glucose hemostasis parameters, 25(OH)VitD, and lipid profile of NAFLD rats.

	Groups			
Items	Control	NAFLD	VitD	NAFLD+ VitD
***ALT*** (***U/L***)	44.80 ± 2.15^b^	59.20 ± 3.02^a^	45.20 ± 1.59^b^	50 ± 2.43^b^
***AST*** (***U/L***)	53.80 ± 2.31^c^	76.80 ± 2.21^a^	56.60 ± 1.11^c^	62.70 ± 0.86^b^
***ALP*** (***U/L***)	333.14 ± 18.73^b^	424.74 ± 14.77^a^	316.88 ± 12.40^b^	342.40 ± 13.42^b^
***Total bilirubin*** (***mg/dL***)	0.22 ± 0.19^b^	0.40 ± 0.02^a^	0.25 ± 0.03^b^	0.38 ± 0.03^a^
***Direct bilirubin*** (***mg/dL***)	0.13 ± 0.03^b^	0.28 ± 0.02^a^	0.13 ± 0.02^b^	0.23 ± 0.02^a^
***Indirect bilirubin*** (***mg/dL***)	0.09 ± 0.02^a^	0.12 ± 0.01^a^	0.12 ± 0.03^a^	0.15 ± 0.02^a^
***Total protein*** (***g/dL***)	9.41 ± 0.20^a^	7.72 ± 0.06^b^	9.49 ± 0.23^a^	8.06 ± 0.15^b^
***Albumin*** (***g/dL***)	4.73 ± 0.26^a^	4.39 ± 0.10^a^	4.33 ± 0.25^a^	4.59 ± 0.10^a^
***Globulin*** (***g/dL***)	4.68 ± 0.12^a^	3.33 ± 0.12^b^	5.17 ± 0.42^a^	3.47 ± 0.18^b^
***Glucose*** (***mg/dL***)	94.55 ± 3.71^c^	141.49 ± 5.01^a^	97.45 ± 1.20^c^	115.87 ± 3.64^b^
***Insulin*** (***μIU/mL***)	5.37 ± 0.12^c^	8.20 ± 0.17^a^	5.40 ± 0.06^c^	6.53 ± 0.58^b^
***HOMA-IR***	1.21 ± 0.04^c^	2.82 ± 0.05^a^	1.27 ± 0.03^c^	1.93 ± 0.13^b^
***25*** (***OH***) ***VitD*** (***ng/mL***)	36.27 ± 1.07^c^	16.05 ± 0.35^d^	150.00 ± 7.22^a^	112.50 ± 7.22^b^
***TG*** (***mg/dL***)	182.78 ± 4.88^c^	262.06 ± 9.51^a^	177.49 ± 6.53^c^	231.70 ± 5.50^b^
***TC*** (***mg/dL***)	182.99 ± 2.46^b^	199.20 ± 3.34^a^	182.59 ± 4.27^b^	186.60 ± 1.29^b^
***VLDL-C*** (***mg/dL***)	36.55 ± 0.98^c^	52.41 ± 1.23^a^	35.49 ± 1.31^c^	46.34 ± 1.10^b^
***LDL-C*** (***mg/dL***)	70.44 ± 4.80^b^	86.54 ± 4.79^a^	70.60 ± 5.50^b^	70.66 ± 5.08^b^
***HDL-c*** (***mg/dL***)	76.00 ± 1.76^a^	60.25 ± 1.24^c^	76.50 ± 1.96^a^	69.60 ± 3.41^b^

Data for (n = 8) are presented as means ± SEM, in which those with different superscripts differed significantly (*p* < 0.05). a is used for the high value, b and c are used for intermediate values between a and d, and d is used for the lower value. *Cont*., control; *NAFLD,* nonalcoholic fatty liver disease; *VitD*, vitamin D; ***ALT***, alanine aminotransferase; ***AST***, aspartate aminotransferase; ***ALP*,** alkaline phosphatase; ***HOMA-IR***: homeostatic model assessment for insulin resistance; ***TG,*** triglyceride; ***TC*,** total cholesterol; ***VLDL-C,*** very-low-density lipoprotein cholesterol; ***LDL-C,*** low-density lipoprotein cholesterol; ***HDL-C,*** high-density lipoprotein cholesterol.

Serum glucose and insulin levels, together with HOMA-IR index, were sustainably (*p* < 0.05) increased, while the serum 25 (OH)VitD level was decreased (*p* < 0.05) significantly in the NAFLD group compared the control group, suggesting disorders of glucose metabolism and a state of insulin resistance. However, VitD treatment markedly (*p* < 0.05) attenuated the high-fat and fructose diet (HFFD)-mediated increase in glucose, insulin, and HOMA-IR values and significantly (*p* < 0.05) improved the serum 25(OH)VitD level in the NAFLD+ VitD group compared to the untreated NAFLD rats (Table 2).

### 3.3 Vitamin D treatment improved the serum lipid profile in NAFLD rats

Table 2 shows that rats in the NAFLD group had significantly (*p* < 0.05) higher TG, TC, VLDL-C, and LDL-C serum levels, while the HDL-C serum level significantly decreased compared with the control rats, indicating impaired lipid homeostasis. Serum levels of TG and VLDL-C notably (*p* < 0.05) decreased, while TC and LDL-C levels almost returned to their basal values, and the HDL-C serum concentration was significantly increased in the NAFLD + VitD group by intramuscular VitD treatment as opposed to the NAFLD group.

### 3.4 Vitamin D improved hepatic antioxidant defense status and serum total antioxidant capacity in NAFLD rats

As shown in Table 3, NAFLD rats exhibited an imbalance in hepatic redox status, as evidenced by a substantial (*p* < 0.05) increment in the hepatic MDA concentration and marked decreases in sTAC, hepatic SOD, GSH, and CAT concentrations compared to the control group. In contrast, VitD effectivity decreased the MDA hepatic concentration and increased hepatic SOD and GSH concentrations in the NAFLD + VitD group compared to the NAFLD group.

**TABLE 3:** Impact of VitD on alterations in the hepatic redox system and serum total antioxidant capacity of NAFLD rats.

	Groups			
Items	Control	NAFLD	VitD	NAFLD+ VitD
***MDA*** (***nmol/g tissue***)	54.58 ± 2.85^b^	76.40 ± 2.48^a^	48.62 ± 3.11^b^	52.49 ± 1.32^b^
***SOD*** (***U/g.tissue***)	478.68 ± 14.54^a^	384.86 ± 11.70^c^	480.26 ± 8.44^a^	436.36 ± 11.58^b^
***GSH*** (***mg/g.tissue***)	5.56 ± 0.29^a^	3.30 ± 0.20^c^	5.57 ± 0.15^a^	4.61 ± 0.16^b^
***CAT*** (***U/g.tissue***)	1.93 ± 0.01^a^	1.75 ± 0.03b	1.90 ± 0.01^a^	1.82 ± 0.03^b^
***sTAC*** (***mM/L***)	1.17 ± 0.15^a^	0.47 ± 0.03^b^	1.05 ± 0.05^a^	0.53 ± 0.06^b^

Data for (n = 8) are presented as means ± SEM, in which those with different superscripts differed significantly (*p* < 0.05). a is used for the high value, b and c are used for intermediate values between a and d, and d is used for the lower value. *Cont*., control; *NAFLD,* nonalcoholic fatty liver disease; *VitD*, vitamin D; ***MDA,*** malondialdehyde; ***SOD,*** superoxide dismutase; **CAT,** catalase; ***GSH,*** reduced glutathione; ***sTAC,*** serum total antioxidant capacity.

### 3.5 Vitamin D treatment modulated the hepatic pro- and antiinflammatory status in NAFLD rats

A significant (*p* < 0.05) increase in the NF-κB level and a sustainable (*p* < 0.05) decrease in the IL-10 level were observed in the liver of NAFLD rats with respect to the control group, indicative of hepatic inflammation. As expected, vitamin D treatment reduced hepatic inflammation and ameliorated the antiinflammatory status as indicated in the NAFLD + VitD group versus the untreated NAFLD group (Table 4).

**TABLE 4:** Impact of Vit D on changes in the hepatic inflammatory status of NAFLD rats.

	Groups			
Items	Control	NAFLD	VitD	NAFLD+ VitD
***NF-κβ*** (***pg/g protein***)	40.00 ± 2.52^c^	474.67 ± 16.38^a^	48.67 ± 4.91^c^	115.33 ± 9.06^b^
***IL-10*** (***pg/g protein***)	143.00 ± 1.53^a^	74.00 ± 6.35^c^	137.64 ± 4.09^a^	121.33 ± 3.76^b^

Data for (n = 8) are presented as means ± SEM, in which those with different superscripts differed significantly (*p* < 0.05). a is used for the high value, b and c are used for intermediate values between a and d, and d is used for the lower value. *Cont*., control; *NAFLD,* nonalcoholic fatty liver disease; *VitD*, vitamin D; ***NF-κβ*,** nuclear factor kappa β; ***IL-10*,** interleukin-10.

### 3.6 Vitamin D improved gene expression in the liver of NAFLD rats

**FIGURE 2:**
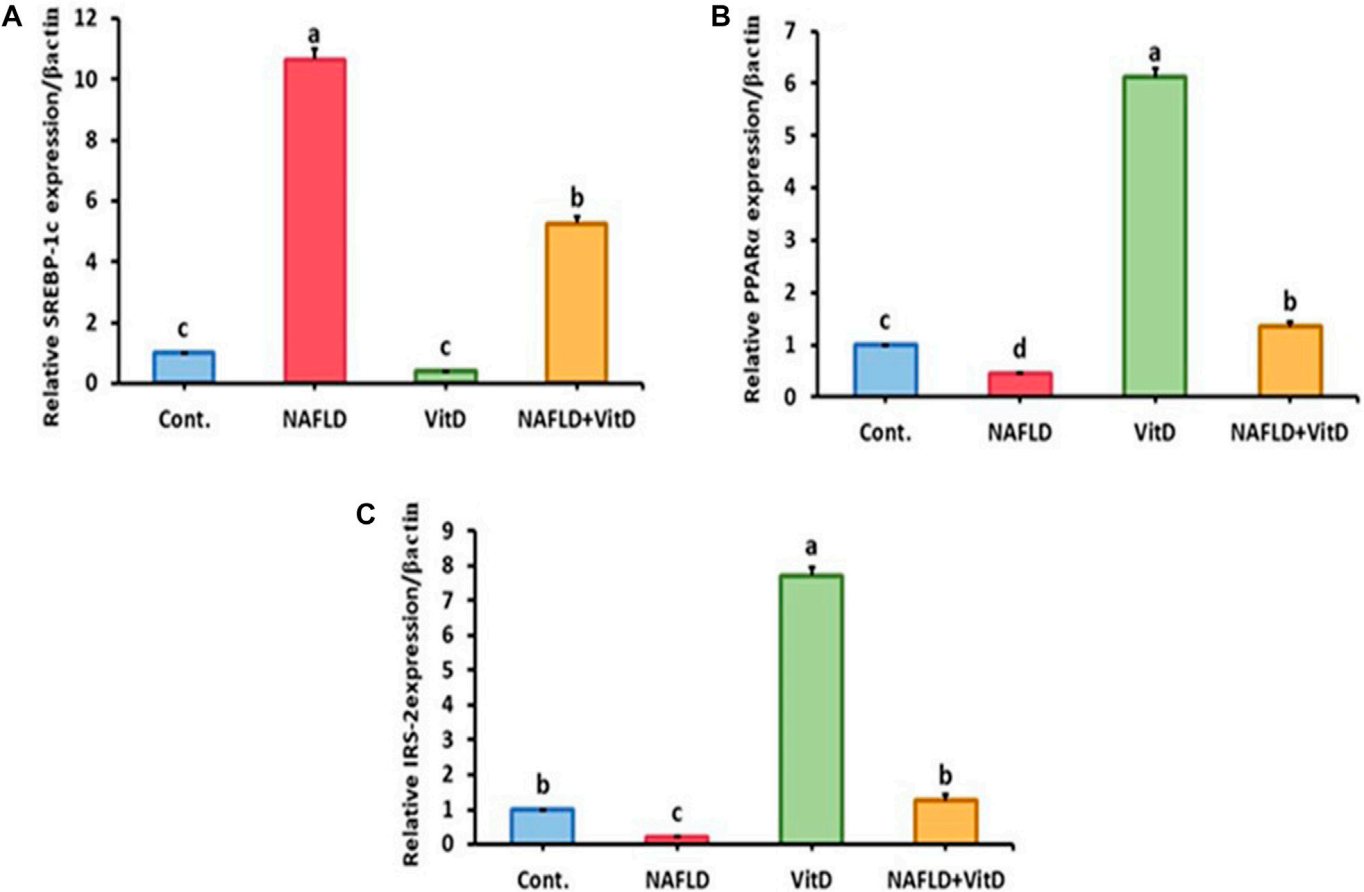
Impact of VitD on the hepatic mRNA expression levels of NAFLD rats. **(A)** SREBP-1-c, **(B)** PPAR-α, and **(C)** IRS-2. Data are shown as means ± SEM, in which those with different superscripts differed significantly (*p* < 0.05). a is used for the high value, b and c are used for intermediate values between a and d, and d is used for the lower value. *Cont*., control; *NAFLD,* nonalcoholic fatty liver disease; and *VitD*, vitamin D. *SREBP-1-c*, sterol regulatory element-binding protein 1-c; *PPAR-α*, peroxisome proliferator-activated receptor alpha; and *RS-2*, insulin receptor substrate-2.

Hepatic gene expression analysis shown in Figure 2 revealed that the hepatic mRNA expression of SREBP-1-c was markedly (*p* < 0.05) upregulated, while hepatic PPAR-α and IRS-2 mRNA expressions levels were markedly (*p* < 0.05) downregulated in the NAFLD rats relative to the control group. Moreover, VitD treatment significantly (*p* < 0.05) abolished the HFFD-mediated downregulation of SREBP-1-c and significantly promoted the mRNA expression of PPAR-α and IRS-2 in the liver of the NAFLD + VitD group versus the NAFLD group.

### 3.7 Vitamin D ameliorated the hepatic histopathological changes observed in NAFLD rats

**FIGURE 3:**
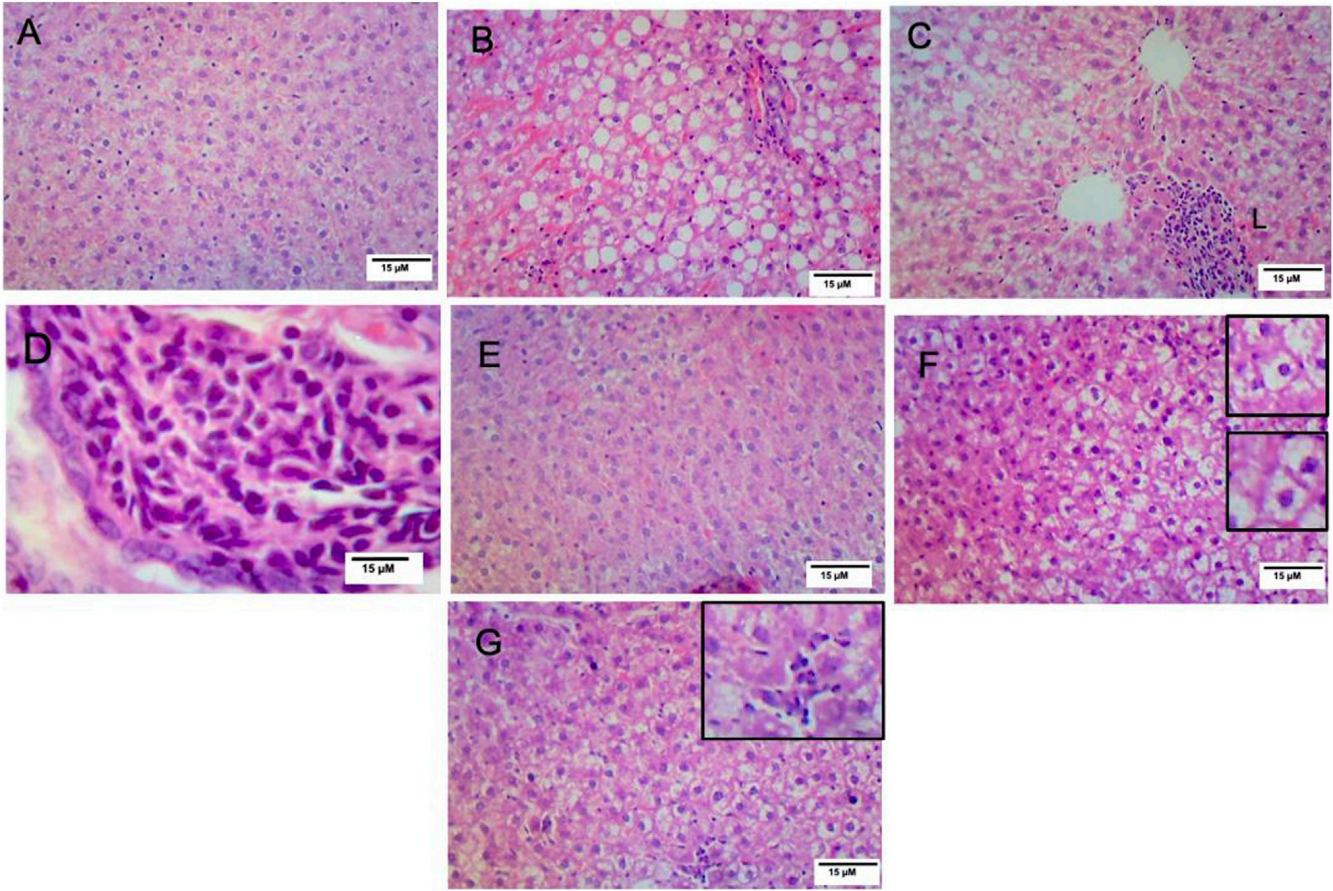
Effect of VitD on hepatic histopathological alterations in NAFLD rats (H&E staining). **(A,E)** Micrographs of the control group and vitamin D-treated group showing normal liver histology (10x). **(B)** Micrograph of the NAFLD group showing microvesicular steatosis and ballooning degeneration (10x). **(C)** Micrograph of the NAFLD group showing lobular inflammation consisting mainly of polymorphonuclear cell infiltration (10x). **(D)** Micrograph of the NAFLD group showing periportal microgranuloma (40x). **(F)** Micrograph of the NAFLD + VitD group showing decreased microvascular steatosis (bottom inset) and hepatocyte ballooning injury (top inset) (10x) compared to the NAFLD group. **(G)** Micrograph of the NAFLD + VitD group showing mild lobular inflammation (inset) (10x).

Histopathological analysis of the hepatic sections revealed that the livers of the control and VitD-treated rats had normal tissue architecture without any detected pathological changes (Figures 3A, E). On the contrary, Table 5 shows that the liver of the NAFLD rats showed parenchyma with intracytoplasmic fat vacuoles and a centrally located nucleus indicating microvesicular fatty changes. The hepatic parenchyma also showed ballooning degeneration and lobular inflammation as opposed to the negative control group (Figures 3B, C). In addition, the liver of the NAFLD rats showed periportal microgranuloma, which can be defined as aggregates of epithelioid cells and other inflammatory cells, including lymphocytes. On the other hand, VitD effectively ameliorated these histopathological abnormalities in the NAFLD + VitD group, as evidenced by a decrease in the observed hepatic microvesicular fatty changes and inflammatory infiltrates when compared to the NAFLD group (Figures 3F, G).

**TABLE 5:** Hepatic scoring and histopathological changes in all experimental groups.

Item	Definition	Score	Control	NAFLD	VitD	NAFLD + VitD
**Steatosis grade**	Low- to medium-power evaluation of parenchymal involvement by steatosis					
	<5%	0	+		+	
	5%–33%	1				+
	>33%–66%	2		+		
	“66%>	3				
**Location**	Predominant distribution pattern					
	Zone 3 or not present	0	+		+	
	Zone 1	1				
	A zonal	2				
	Panacinar	3		+		+
**Microvesicular steatosis**	Contiguous patches					
	Not present	0	+		+	
	Present	1		+		+
**Fibrosis stage**	None	0	+	+	+	+
**Lobular inflammation**	Overall assessment of all inflammatory foci					
	No foci	0	+		+	
	<2 foci per 200X field	1				+
	2–4 foci per 200X field	2		+		
	>4 foci per 200X field	3				
**Portal inflammation**	Assessed from low magnification					
	None to minimal	0	+		+	+
	Greater than minimal	1		+		
**Microgranulomas**	Small aggregates of macrophages					
	Absent	0	+		+	
	Present	1		+		+
**Liver cell injury Ballooning**	None	0	+		+	
	Few balloon cells	1				
	Many cells/prominent ballooning	2		+		+
**Acidophil bodies**	None to rare	0	+	+	+	+
	Many	1				
**Pigmented macrophages**	None to rare	0	+	+	+	+
	Many	1				
**Megamitochondria**	None to rare	0	+		+	
	Many	1		+		+
**Other findings**						
**Mallory’s hyaline**	Visible on routine stains					
	None to rare	0	+		+	
	Many	1		+		+
**Glycogenated nuclei**	Contiguous patches					
	None to rare	0	+	+	+	+
	Many	1				

**FIGURE 4:**
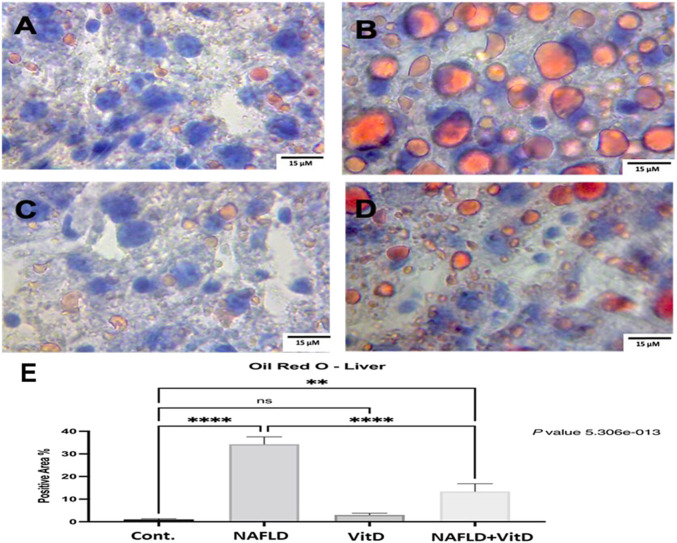
Photomicrographs of Oil Red O-stained hepatic sections (40x). **(A,C)** Cont. group and vitamin D-treated group: showing normal liver histology. **(B)** NAFLD group: showing widespread lipid droplets of variable size occupying the cytoplasm of hepatocytes. **(D)** NAFLD + VitD group: showing decreased lipid deposits compared with the NAFLD group. **(E)** Mean positive area percentage ±SEM in each group. Data were analyzed using one-way ANOVA, followed by Tukey’s test for group comparison. ns = not significant. * = *p* value ≤ 0.05. ** = *p* value ≤ 0.01. *** = *p* value ≤ 0.001. **** = *p* value ≤ 0.0001.

Figure 4 shows photomicrographs of ORO-stained hepatic sections. Histology of the liver was normal in the control and VitD groups (Figures 4A, C), respectively. In the NAFLD group, the liver showed widespread lipid droplets of varied sizes occupying the cytoplasm of hepatocytes (Figure 4B). As expected, the liver of the NAFLD + VitD group had a fewer number of lipid droplets than the NAFLD group (Figures 4D, E).

## 4 Discussion

Nonalcoholic fatty liver disease is a common liver disorder that can range from asymptomatic steatosis to inflammation and fibrosis, which may lead to hepatic cirrhosis and carcinoma (Katsiki et al., 2016). Routine treatments for NAFLD include lifestyle adjustment and surgical therapy; however, the most effective therapeutic regimens for NAFLD are still under study (Liu et al., 2020). Nowadays, the various functions of VitD are known due to its immunomodulatory, antiinflammatory, and insulin-sensitizing properties (Barchetta et al., 2020). Therefore, VitD could be effectively used as a therapy for NAFLD (Eliades and Spyrou, 2015). From this point of view, this study was conducted to explore the possible protective effects of VitD administration against NAFLD caused by a high-fat and fructose diet (HFFD).

In the current study, the HFFD caused a marked increase in the body weight of rats, which may be attributed to the ability of fructose to promote leptin resistance and serum ghrelin levels (Muriel et al., 2021). Vitamin D could not modulate the increase in body weight induced by the HFFD that was previously recorded in rats fed an obesogenic diet and treated with vitamin D (Mazzone et al., 2018; Shojaei Zarghani et al., 2018; Al-Badarein and Ahmad, 2021).

Evaluation of the serum activity of ALT is considered a valuable tool for the detection of NAFLD. In our study, liver function biomarkers, including ALT, AST, ALP, and total and direct bilirubin, were markedly increased in the NAFLD group, indicating hepatocellular damage. This may be due to the lipogenic potential of fructose as it increases hepatic *de novo* lipogenesis (DNL) at multiple levels, leading to intrahepatic fat accumulation (Mouzaki and Allard, 2012; Charrez et al., 2015; Jin and Vos, 2015; Softic et al., 2016). This was confirmed histopathologically in our work with H&E and ORO staining. Administration of VitD had a hepatoprotective effect, as detected by lowering the elevated liver enzymes activities (Han et al., 2015; Zhu et al., 2017), which was confirmed histopathologically by a significant reduction in the hepatic lipid burden. The same findings were reported previously by Shojaei Zarghani et al. (2018) in rats fed an HFFD and treated with a vitamin D and calcium combination. The prevention of intrahepatic fat accumulation and the reduction in hepatic cholesterol content may be the cause of the reduction in the hepatic lipid burden (Mazzone et al., 2018; Shojaei Zarghani et al., 2018). Hence, VitD has been reported to downregulate SREBP-1-c and stimulate PPAR-α (Yin et al., 2012).

In the current study, the NAFLD group showed glucose intolerance and impaired insulin sensitivity, which is consistent with the findings obtained by Geetha et al. (2014). This may be a result of high fructose consumption, as it has been reported that fructose-1-phosphate activates mitogen-activated protein kinase, which phosphorylates insulin receptor substrate 1 at the serine residue, leading to hepatic insulin resistance (IR) (Lim et al., 2010; Chang et al., 2014). In addition, fructose itself promotes forkhead box protein O1 synthesis in the liver, leading to an increase in gluconeogenesis, which, together with IR, leads to hyperglycemia and glucose intolerance (Mouzaki and Allard, 2012). In contrast, VitD administration improved glucose hemostasis in the NAFLD group (Sergeev and Song, 2014). This improvement could be attributed to the ability of calcitriol to activate AMPK via the calcium/calmodulin protein kinase beta pathway, leading to attenuating gluconeogenesis and promoting glycolysis (Leung, 2016; Szymczak-Pajor et al., 2020). Additionally, Sung et al. (2012) claimed that VitD can promote insulin action either directly by stimulating insulin receptor expression or indirectly through the regulation of Ca^++^ levels.

Since NAFLD is associated with lipid disorders, serum lipid profile parameters were investigated. The NAFLD group showed elevations in the serum concentration of TG, TC, VLDL-C, and LDL-C but lower serum HDL-C concentrations. This impairment may be contributed to increase DNL induced by fructose consumption, leading to the formation of fatty acids to produce hepatic TG. The increased hepatic lipid content leads to a decrease in intracellular apo-B degradation and increased production and secretion of VLDL-C (Stanhope and Havel, 2010). On the other hand, the decreased HDL blood level was contributed to the decreased post-heparin plasma LPL/HL ratio and the increased VLDL levels (Mooradian et al., 2008). Concerning the impact of VitD on lipid profile parameters, it ameliorated lipid levels in the serum, which may refer to its ability to promote calcium absorption from the intestine. This calcium forms insoluble soap with fatty acids, especially saturated fatty acids, resulting in increased fecal fat excretion and a decrease in its digestibility (Christensen et al., 2009; Subih et al., 2018). It may also increase lipoprotein lipase enzymes, promote the formation of high-density lipoprotein cholesterol particles, and regulate serum apo-lipoprotein A-1 levels (Hassan et al., 2020; Melguizo-Rodríguez et al., 2021). Our result was previously reported by Farhangi et al. (2017)and El-Sherbiny et al. (2018). In contrast, Shojaei Zarghani et al. (2018) reported insignificant changes in the serum level of TG, TC, and LDL in groups fed with an HFFD and treated with a vitamin D and calcium combination.

Hepatic redox imbalance has been known to be involved in the development and progression of NAFLD from simple steatosis to a more severe form (Spahis et al., 2016). Our data indicated that NAFLD rats had increased hepatic MDA and decreased hepatic antioxidant enzymes and GSH, which was previously reported by Nasri et al. (2015). This may be due to increased superoxide and hydrogen peroxide ions from HFFD feeding (Jarukamjorn et al., 2016). In addition, the antioxidant potential of cells decreases when the excess fructose activates DNL, leading to a reduction in NADPH (Charrez et al., 2015). In agreement with Zhu et al. (2017), our data demonstrated that VitD improved hepatic redox status. The antioxidant effect of VitD may be associated with the inhibition of NADPH oxidase and the enhancement of nuclear factor erythroid 2-related factor 2 nuclear translocation (Nakai et al., 2013; Zhu et al., 2017). Additionally, VitD enhances the synthesis of metal ion protein carriers. These metal ions are critical for the action of various enzymes such as antioxidant enzymes (Hassan et al., 2020).

Our data revealed that NAFLD rats showed an increase in hepatic NF-κB and a decline in the IL-10 levels. There are many potential cellular mechanisms leading to activating inflammatory signaling in NAFLD as a high-caloric diet activates the IKK/NF-κB pathway in adipocytes, hepatocytes, and associated macrophages. IKK activation leads to NF-κB translocation and increased expression of numerous markers and potential mediators of inflammation (Pradhan, 2007). Therefore, the activation of the NF-κB signaling pathway as the most enriched pathway was associated with hepatic inflammation and resulted in an increase in NF-κB expression in NAFLD (Sangouni et al., 2019; Zhao et al., 2022). Meanwhile, the decreased IL-10 levels are linked to T-helper-2 malfunctions (Cano Barquilla et al., 2014). Our findings were previously recorded by Theodoro et al. (2021). On the contrary, VitD administration reduced hepatic inflammation, which was confirmed in our histopathological results by decreasing inflammatory cell infiltration. This could be due to the ability of calcitriol to interfere with NF-κB via increasing the expression of the inhibitory protein (IκB) in peripheral blood mononuclear cells and reducing the nuclear translocation of the NF-κB subunit p65 (Krishnan and Feldman, 2011; Chen et al., 2020). Moreover, the IL-10 hepatic level was significantly enhanced upon VitD treatment, which is in agreement with a prior report (Refaat et al., 2021). This could be attributed to enhancing T-helper-2 cell differentiation and overproduction of antiinflammatory cytokines such as IL-10 after VitD administration (Bishop et al., 2021; Sharma et al., 2021).

To further confirm the hepatic lipid burden in our NAFLD model with a decrease in its oxidation, we performed qualitative real-time PCR for analyzing the hepatic expression of lipid metabolic genes including SREBP-1-c and PPAR-α. The results revealed disturbances in the expression of both genes in the NAFLD group including an increase in SREBP-1-c expression and a decrease in PPAR-α expression, which was previously observed by Wang et al. (2022). The explanation for the observed changes in the hepatic SREBP-1-c and PPAR-α mRNA levels is provided by Nagai et al. (2002), who observed that fructose can stimulate SREBP-1-c but suppress PPAR-α expression levels in rat liver. VitD administration resulted in reverse changes in both genes’ expression in the liver of the NAFLD group. This may be related to the ability of VitD to interfere with the activation of SREBP-1-c by enhancing the ubiquitin-mediated degradation of the SREBP cleavage-activating protein (Asano et al., 2017). Similar results were previously observed in male rats fed with an HFD and treated with calcitriol at doses 5 μg/kg B.W, I/P, and 5 ng/g B.W, I/M, twice per week, respectively (Yin et al., 2012; Kong et al., 2014).

The impairment of the insulin signal and insulin resistance was further confirmed in our NAFLD model by a significant decrease in the hepatic expression of IRS-2. This may refer to the ability of SREBP-1-c to suppress IRS-2 promoter activity by competing with its transactivator (Ide et al., 2004). Several studies confirmed that hepatic IRS-2 mRNA expression was downregulated in HFD-fed rodent models (Xing et al., 2011; Qiu et al., 2015; Yang et al., 2019). Contrariwise, vitamin D administration enhanced hepatic IRS-2 expression in NAFLD rats. Our result is in parallel with that obtained by Szymczak-Pajor et al. (2020), who speculated that the active form of VitD enhances the transcriptional activation of the IR gene, which improves insulin signaling.

## 5 Conclusion

This study clarified that an HFFD led to a substantial increase in body weight and hepatic injury indices, along with disorders in glucose hemostasis and lipid metabolism. In addition, hepatic inflammation and oxidative damage occurred, which led to the development and progression of NAFLD. We also concluded that VitD may protect against HFFD-induced NAFLD through its antioxidant and antiinflammatory effects. In addition, VitD had ameliorative effects on glucose hemostasis and lipid profile.

## Data Availability

The original contributions presented in the study are included in the article/Supplementary Material; further inquiries can be directed to the corresponding author.

## References

[B1] Al-Badarein B. O. Ahmad M. N. (2021). Body weight, insulin resistance, and inflammatory biomarkers in rats fed normal-fat, high-fat, and ketogenic diets supplemented with vitamin D. J. Agric. Sci. 17 (1), 1–16. 10.35516/jjas.v17i1.64

[B2] Asano L. Watanabe M. Ryoden Y. Usuda K. Yamaguchi T. Khambu B. (2017). Vitamin D metabolite, 25-hydroxyvitamin D, regulates lipid metabolism by inducing degradation of SREBP/SCAP. Cell Chem. Biol. 24 (2), 207–217. 10.1016/j.chembiol.2016.12.017 28132894

[B3] Banni M. Messaoudi I. Said L. El Heni J. Kerkeni A. Said K. (2010). Metallothionein gene expression in liver of rats exposed to cadmium and supplemented with zinc and selenium. Archives Environ. Contam. Toxicol. 59 (3), 513–519. 10.1007/s00244-010-9494-5 20238111

[B4] Barchetta I. Cimini F. A. Cavallo M. G. (2020). Vitamin D and metabolic dysfunction-associated fatty liver disease (MAFLD): An update. Nutrients 12 (11), 3302. 10.3390/nu12113302 33126575 PMC7693133

[B5] Barchetta I. Cimini F. A. Cavallo M. G. (2017). Vitamin D supplementation and non-alcoholic fatty liver disease: Present and future. Nutrients 9 (9), 1015. 10.3390/nu9091015 28906453 PMC5622775

[B6] BaSalamah M. A. Abdelghany A. H. El-Boshy M. Ahmad J. Idris S. Refaat B. (2018). Vitamin D alleviates lead induced renal and testicular injuries by immunomodulatory and antioxidant mechanisms in rats. Sci. Rep. 8 (1), 4853. 10.1038/s41598-018-23258-w 29556070 PMC5859277

[B7] Bishop L. E. Ismailova A. Dimeloe S. Hewison M. White J. H. (2021). Vitamin D and immune regulation: Antibacterial, antiviral, anti‐inflammatory. JBMR plus 5 (1), e10405. 10.1002/jbm4.10405 32904944 PMC7461279

[B8] Bradford M. M. (1976). A rapid and sensitive method for the quantitation of microgram quantities of protein utilizing the principle of protein-dye binding. Anal. Biochem. 72 (1), 248–254. 10.1006/abio.1976.9999 942051

[B9] Cano Barquilla P. Pagano E. S. Jiménez‐Ortega V. Fernández‐Mateos P. Esquifino A. I. Cardinali D. P. (2014). Melatonin normalizes clinical and biochemical parameters of mild inflammation in diet‐induced metabolic syndrome in rats. J. pineal Res. 57 (3), 280–290. 10.1111/jpi.12168 25113124

[B10] Chang W.-C. Jia H. Aw W. Saito K. Hasegawa S. Kato H. (2014). Beneficial effects of soluble dietary Jerusalem artichoke (Helianthus tuberosus) in the prevention of the onset of type 2 diabetes and non-alcoholic fatty liver disease in high-fructose diet-fed rats. Br. J. Nutr. 112 (5), 709–717. 10.1017/S0007114514001421 24968200

[B11] Charrez B. Qiao L. Hebbard L. (2015). The role of fructose in metabolism and cancer. Hormone Mol. Biol. Clin. Investigation 22 (2), 79–89. 10.1515/hmbci-2015-0009 25965509

[B12] Chen J. Tang Z. Slominski A. T. Li W. Żmijewski M. A. Liu Y. (2020). Vitamin D and its analogs as anticancer and anti-inflammatory agents. Eur. J. Med. Chem. 207, 112738. 10.1016/j.ejmech.2020.112738 32829183

[B13] Christensen R. Lorenzen J. K. Svith C. R. Bartels E. Melanson E. Saris W. (2009). Effect of calcium from dairy and dietary supplements on faecal fat excretion: A meta‐analysis of randomized controlled trials. Obes. Rev. 10 (4), 475–486. 10.1111/j.1467-789X.2009.00599.x 19493303

[B14] Ding L. Li J. Song B. Xiao X. Huang W. Zhang B. (2014). Andrographolide prevents high-fat diet–induced obesity in C57bl/6 mice by suppressing the sterol regulatory element-binding protein pathway. J. Pharmacol. Exp. Ther. 351 (2), 474–483. 10.1124/jpet.114.217968 25204338

[B15] El-Sherbiny M. Eldosoky M. El-Shafey M. Othman G. Elkattawy H. A. Bedir T. (2018). Vitamin D nanoemulsion enhances hepatoprotective effect of conventional vitamin D in rats fed with a high-fat diet. Chemico-Biological Interact. 288, 65–75. 10.1016/j.cbi.2018.04.010 29653100

[B16] Eliades M. Spyrou E. (2015). Vitamin D: A new player in non-alcoholic fatty liver disease? World J. gastroenterology WJG 21 (6), 1718–1727. 10.3748/wjg.v21.i6.1718 PMC4323447 25684936

[B17] Faraji S. Alizadeh M. (2020). Mechanistic effects of vitamin D supplementation on metabolic syndrome components in patients with or without vitamin D deficiency. J. Obes. Metab. Syndr. 29 (4), 270–280. 10.7570/jomes20003 32747610 PMC7789020

[B18] Farhangi M. A. Nameni G. Hajiluian G. Mesgari-Abbasi M. (2017). Cardiac tissue oxidative stress and inflammation after vitamin D administrations in high fat-diet induced obese rats. BMC Cardiovasc. Disord. 17 (1), 161. 10.1186/s12872-017-0597-z 28629326 PMC5477304

[B19] Friedewald W. T. Levy R. I. Fredrickson D. S. (1972). Estimation of the concentration of low-density lipoprotein cholesterol in plasma, without use of the preparative ultracentrifuge. Clin. Chem. 18 (6), 499–502. 10.1093/clinchem/18.6.499 4337382

[B20] Geetha R. Yogalakshmi B. Sreeja S. Bhavani K. Anuradha C. V. (2014). Troxerutin suppresses lipid abnormalities in the heart of high-fat–high-fructose diet-fed mice. Mol. Cell. Biochem. 387 (1), 123–134. 10.1007/s11010-013-1877-2 24173620

[B21] Green H. Kehinde O. (1974). Sublines of mouse 3T3 cells that accumulate lipid. Cell 1 (3), 113–116. 10.1016/0092-8674(74)90126-3

[B22] Han H. Cui M. You X. Chen M. Piao X. Jin G. (2015). A role of 1,25(OH)2D3 supplementation in rats with nonalcoholic steatohepatitis induced by choline-deficient diet. Nutr. Metabolism Cardiovasc. Dis. 25 (6), 556–561. 10.1016/j.numecd.2015.02.011 25843661

[B23] Hassan F. El-Said E.-S. E.-S. El-sayed G. R. El-Sayed S. A. E.-S. Awadin W. F. (2020). Nano-particles of trace minerals in poultry nutrition: Potential applications and future prospects. Comp. Clin. Pathol. 29 (3), 591–612. 10.1007/s12011-019-01862-9 31473896

[B24] Ide T. Shimano H. Yahagi N. Matsuzaka T. Nakakuki M. Yamamoto T. (2004). SREBPs suppress IRS-2-mediated insulin signalling in the liver. Nat. Cell Biol. 6 (4), 351–357. 10.1038/ncb1111 15048126

[B25] Jarukamjorn K. Jearapong N. Pimson C. Chatuphonprasert W. (2016). A high-fat, high-fructose diet induces antioxidant imbalance and increases the risk and progression of nonalcoholic fatty liver disease in mice. Scientifica 2016, 5029414. 10.1155/2016/5029414 27019761 PMC4785277

[B26] Jin R. Vos M. B. (2015). Fructose and liver function – is this behind nonalcoholic liver disease? Curr. Opin. Clin. Nutr. Metabolic Care 18 (5), 490–495. 10.1097/MCO.0000000000000203 26203597

[B27] Kanuri G. Landmann M. Priebs J. Spruss A. Löscher M. Ziegenhardt D. (2016). Moderate alcohol consumption diminishes the development of non-alcoholic fatty liver disease (NAFLD) in ob/ob mice. Eur. J. Nutr. 55 (3), 1153–1164. 10.1007/s00394-015-0929-7 26003186

[B28] Katsiki N. Mikhailidis D. P. Mantzoros C. S. (2016). Non-alcoholic fatty liver disease and dyslipidemia: An update. Metabolism 65 (8), 1109–1123. 10.1016/j.metabol.2016.05.003 27237577

[B29] Kaufmann B. Reca A. Wang B. Friess H. Feldstein A. E. Hartmann D. (2021). Mechanisms of nonalcoholic fatty liver disease and implications for surgery. Langenbeck's Archives Surg. 406 (1), 1–17. 10.1007/s00423-020-01965-1 PMC7870612 32833053

[B30] Kleiner D. E. Brunt E. M. Van Natta M. Behling C. Contos M. J. Cummings O. W. (2005). Design and validation of a histological scoring system for nonalcoholic fatty liver disease. Hepatology 41 (6), 1313–1321. 10.1002/hep.20701 15915461

[B31] Kong M. Zhu L. Bai L. Zhang X. Chen Y. Liu S. (2014). Vitamin D deficiency promotes nonalcoholic steatohepatitis through impaired enterohepatic circulation in animal model. Am. J. Physiology-Gastrointestinal Liver Physiology 307 (9), G883–G893. 10.1152/ajpgi.00427.2013 PMC4216990 25214402

[B32] Krishnan A. V. Feldman D. (2011). Mechanisms of the anti-cancer and anti-inflammatory actions of vitamin D. Annu. Rev. Pharmacol. Toxicol. 51, 311–336. 10.1146/annurev-pharmtox-010510-100611 20936945

[B33] Leung P. S. (2016). The potential protective action of vitamin D in hepatic insulin resistance and pancreatic islet dysfunction in type 2 diabetes mellitus. Nutrients 8 (3), 147. 10.3390/nu8030147 26959059 PMC4808876

[B34] Lim J. S. Mietus-Snyder M. Valente A. Schwarz J.-M. Lustig R. H. (2010). The role of fructose in the pathogenesis of NAFLD and the metabolic syndrome. Nat. Rev. Gastroenterology Hepatology 7 (5), 251–264. 10.1038/nrgastro.2010.41 20368739

[B35] Liu Y. Wang M. Xu W. Zhang H. Qian W. Li X. (2020). Active vitamin D supplementation alleviates initiation and progression of nonalcoholic fatty liver disease by repressing the p53 pathway. Life Sci. 241, 117086. 10.1016/j.lfs.2019.117086 31756344

[B36] Livak K. J. Schmittgen T. D. (2001). Analysis of relative gene expression data using real-time quantitative PCR and the 2(-Delta Delta C(T)) Method. Methods 25 (4), 402–408. 10.1006/meth.2001.1262 11846609

[B37] Loomba R. Sanyal A. J. (2013). The global NAFLD epidemic. Nat. Rev. Gastroenterology Hepatology. 10 (11), 686–690. 10.1038/nrgastro.2013.171 24042449

[B38] Matthews D. R. Hosker J. P. Rudenski A. S. Naylor B. A. Treacher D. F. Turner R. C. (1985). Homeostasis model assessment: Insulin resistance and β-cell function from fasting plasma glucose and insulin concentrations in man. Diabetologia 28 (7), 412–419. 10.1007/BF00280883 3899825

[B39] Mazzone G. Morisco C. Lembo V. D’Argenio G. D’Armiento M. Rossi A. (2018). Dietary supplementation of vitamin D prevents the development of Western diet-induced metabolic, hepatic and cardiovascular abnormalities in rats. United Eur. Gastroenterology J. 6 (7), 1056–1064. 10.1177/2050640618774140 PMC6137584 30228894

[B40] Melguizo-Rodríguez L. Costela-Ruiz V. J. García-Recio E. De Luna-Bertos E. Ruiz C. Illescas-Montes R. (2021). Role of vitamin D in the metabolic syndrome. Nutrients 13 (3), 830. 10.3390/nu13030830 33802330 PMC7999005

[B41] Mooradian A. D. Haas M. J. Wehmeier K. R. Wong N. C. W. (2008). Obesity-related changes in high-density lipoprotein metabolism. Obesity 16 (6), 1152–1160. 10.1038/oby.2008.202 18388903

[B42] Mouzaki M. Allard J. P. (2012). The role of nutrients in the development, progression, and treatment of nonalcoholic fatty liver disease. J. Clin. Gastroenterology 46 (6), 457–467. 10.1097/MCG.0b013e31824cf51e 22469640

[B43] Muriel P. López-Sánchez P. Ramos-Tovar E. (2021). Fructose and the liver. Int. J. Mol. Sci. 22 (13), 6969. 10.3390/ijms22136969 34203484 PMC8267750

[B44] Nagai Y. Nishio Y. Nakamura T. Maegawa H. Kikkawa R. Kashiwagi A. (2002). Amelioration of high fructose-induced metabolic derangements by activation of PPARalpha. Am. J. Physiology-Endocrinology Metabolism 282 (5), E1180–E1190. 10.1152/ajpendo.00471.2001 11934685

[B45] Nakai K. Fujii H. Kono K. Goto S. Kitazawa R. Kitazawa S. (2013). Vitamin D activates the nrf2-keap1 antioxidant pathway and ameliorates nephropathy in diabetic rats. Am. J. Hypertens. 27 (4), 586–595. 10.1093/ajh/hpt160 24025724

[B46] Nasri R. Abdelhedi O. Jemil I. Daoued I. Hamden K. Kallel C. (2015). Ameliorating effects of goby fish protein hydrolysates on high-fat-high-fructose diet-induced hyperglycemia, oxidative stress and deterioration of kidney function in rats. Chemico-Biological Interact. 242, 71–80. 10.1016/j.cbi.2015.08.003 26327248

[B47] Pradhan A. (2007). Obesity, metabolic syndrome, and type 2 diabetes: Inflammatory basis of glucose metabolic disorders. Nutr. Rev. 65 (3), S152–S156. 10.1111/j.1753-4887.2007.tb00354.x 18240540

[B48] Qiu X. Gao D.-H. Xiang X. Xiong Y.-F. Zhu T.-S. Liu L.-G. (2015). Ameliorative effects of lutein on non-alcoholic fatty liver disease in rats. World J. Gastroenterol. 21 (26), 8061–8072. 10.3748/wjg.v21.i26.8061 26185377 PMC4499348

[B49] Rahimi R. S. Landaverde C. (2013). Nonalcoholic fatty liver disease and the metabolic syndrome: Clinical implications and treatment. Nutr. Clin. Pract. 28 (1), 40–51. 10.1177/0884533612470464 23286927

[B50] Refaat B. Abdelghany A. H. Ahmad J. Abdalla O. M. Elshopakey G. E. Idris S. (2021). Vitamin D3 enhances the effects of omega-3 oils against metabolic dysfunction-associated fatty liver disease in rat. BioFactors. 10.1002/biof.1804 34767670

[B51] Ren L. Sun D. Zhou X. Yang Y. Huang X. Li Y. (2019). Chronic treatment with the modified Longdan Xiegan Tang attenuates olanzapine-induced fatty liver in rats by regulating hepatic de novo lipogenesis and fatty acid beta-oxidation-associated gene expression mediated by SREBP-1c, PPAR-alpha and AMPK-alpha. J. Ethnopharmacol. 232, 176–187. 10.1016/j.jep.2018.12.034 30590197

[B52] Sangouni A. A. Ghavamzadeh S. Jamalzehi A. (2019). A narrative review on effects of vitamin D on main risk factors and severity of Non-Alcoholic Fatty Liver Disease. Diabetes & Metabolic Syndrome Clin. Res. Rev. 13 (3), 2260–2265. 10.1016/j.dsx.2019.05.013 31235166

[B53] Sergeev I. N. Song Q. (2014). High vitamin D and calcium intakes reduce diet-induced obesity in mice by increasing adipose tissue apoptosis. Mol. Nutr. Food Res. 58 (6), 1342–1348. 10.1002/mnfr.201300503 24449427

[B54] Sharifi N. Amani R. (2019). Vitamin D supplementation and non-alcoholic fatty liver disease: A critical and systematic review of clinical trials. Crit. Rev. food Sci. Nutr. 59 (4), 693–703. 10.1080/10408398.2017.1389693 29035092

[B55] Sharma K. Zajc I. Žiberna L. (2021). Dietary vitamin D equilibrium in serum ameliorates direct bilirubin associated diabetes mellitus. Chemico-Biological Interact. 337, 109399. 10.1016/j.cbi.2021.109399 33503443

[B56] Shin S.-K. Cho S.-J. Jung U. J. Ryu R. Choi M.-S. (2016). Phlorizin supplementation attenuates obesity, inflammation, and hyperglycemia in diet-induced obese mice fed a high-fat diet. Nutrients 8 (2), 92. 10.3390/nu8020092 26891322 PMC4772055

[B57] Shojaei Zarghani S. Soraya H. Alizadeh M. (2018). Calcium and vitamin D 3 combinations improve fatty liver disease through AMPK-independent mechanisms. Eur. J. Nutr. 57, 731–740. 10.1007/s00394-016-1360-4 27988847

[B58] Softic S. Cohen D. E. Kahn C. R. (2016). Role of dietary fructose and hepatic de novo lipogenesis in fatty liver disease. Dig. Dis. Sci. 61 (5), 1282–1293. 10.1007/s10620-016-4054-0 26856717 PMC4838515

[B59] Souza M. RdA. Diniz M. d. F. F. d. M. Medeiros-Filho J. E. M. d. Araújo M. S. T. d. (2012). Metabolic syndrome and risk factors for non-alcoholic fatty liver disease. Arq. Gastroenterol. 49, 89–96. 10.1590/s0004-28032012000100015 22481692

[B60] Spahis S. Delvin E. Borys J.-M. Levy E. (2016). Oxidative stress as a critical factor in nonalcoholic fatty liver disease pathogenesis. Antioxidants Redox Signal. 26 (10), 519–541. 10.1089/ars.2016.6776 27452109

[B61] Stanhope K. L. Havel P. J. (2010). Fructose consumption: Recent results and their potential implications. Ann. N. Y. Acad. Sci. 1190 (1), 15–24. 10.1111/j.1749-6632.2009.05266.x 20388133 PMC3075927

[B62] Subih H. Al-Tamimi H. Hamdan H. Bawadi H. Janakat S. (2018). Decreased weight gain and enhanced serum biochemical parameters in rats after vitamin D and Ca supplementation. Malays. J. Nutr. 24 (2).

[B63] Sung C.-C. Liao M.-T. Lu K.-C. Wu C.-C. (2012). Role of vitamin D in insulin resistance. J. Biomed. Biotechnol. 2012, 634195. 10.1155/2012/634195 22988423 PMC3440067

[B64] Szymczak-Pajor I. Drzewoski J. Śliwińska A. (2020). The molecular mechanisms by which vitamin D prevents insulin resistance and associated disorders. Int. J. Mol. Sci. 21 (18), 6644. 10.3390/ijms21186644 32932777 PMC7554927

[B65] Theodoro J. M. V. Martinez O. D. M. Grancieri M. Toledo R. C. L. Dias Martins A. M. Dias D. M. (2021). Germinated millet flour (Pennisetum glaucum (L) R Br) reduces inflammation, oxidative stress, and liver steatosis in rats fed with high-fat high-fructose diet. J. Cereal Sci. 99, 103207. 10.1016/j.jcs.2021.103207

[B66] Wang J.-H. Hwang S.-J. Lim D.-W. Son C.-G. (2022). Cynanchum atratum alleviates non-alcoholic fatty liver by balancing lipogenesis and fatty acid oxidation in a high-fat, high-fructose diet mice model. Cells 11 (1), 23. 10.3390/cells11010023 PMC8750091 35011585

[B67] Xing L.-J. Zhang L. Liu T. Hua Y.-Q. Zheng P.-Y. Ji G. (2011). Berberine reducing insulin resistance by up-regulating IRS-2 mRNA expression in nonalcoholic fatty liver disease (NAFLD) rat liver. Eur. J. Pharmacol. 668 (3), 467–471. 10.1016/j.ejphar.2011.07.036 21839075

[B68] Yang P. Liang Y. Luo Y. Li Z. Wen Y. Shen J. (2019). Liraglutide ameliorates nonalcoholic fatty liver disease in diabetic mice via the IRS2/PI3K/Akt signaling pathway. Diabetes Metab. Syndr. Obes. 12, 1013–1021. 10.2147/DMSO.S206867 31308717 PMC6614831

[B69] Yin Y. Yu Z. Xia M. Luo X. Lu X. Ling W. (2012). Vitamin D attenuates high fat diet–induced hepatic steatosis in rats by modulating lipid metabolism. Eur. J. Clin. Investigation 42 (11), 1189–1196. 10.1111/j.1365-2362.2012.02706.x 22958216

[B70] Zhao W. Guo M. Feng J. Gu Z. Zhao J. Zhang H. (2022). Myristica fragrans extract regulates gut microbes and metabolites to attenuate hepatic inflammation and lipid metabolism disorders via the AhR–FAS and NF-κB signaling pathways in mice with non-alcoholic fatty liver disease. Nutrients 14 (9), 1699. 10.3390/nu14091699 35565666 PMC9104743

[B71] Zhu C.-g. Liu Y.-x. Wang H. Wang B.-p. Qu H.-q. Wang B.-l. (2017). Active form of vitamin D ameliorates non-alcoholic fatty liver disease by alleviating oxidative stress in a high-fat diet rat model. Endocr. J. 64 (7), 663–673. 10.1507/endocrj.EJ16-0542 28539530

[B72] Zivkovic A. M. German J. B. Sanyal A. J. (2007). Comparative review of diets for the metabolic syndrome: Implications for nonalcoholic fatty liver disease. Am. J. Clin. Nutr. 86 (2), 285–300. 10.1093/ajcn/86.2.285 17684197

